# Unusual presentation of dermatofibroma on the face: Case report

**DOI:** 10.1002/ccr3.2066

**Published:** 2019-02-19

**Authors:** Maha AlQusayer, Mei AlQusayer, Salim Alkeraye

**Affiliations:** ^1^ College of Medicine Princess Nourah University Riyadh Saudi Arabia; ^2^ Department of Dermatology, College of Medicine King Khalid University Hospital Riyadh Saudi Arabia

**Keywords:** benign fibrous histiocytoma, dermatofibroma, face

## Abstract

Dermatofibroma (DF; Benign Fibrous Histiocytoma) seldom presents on the face. We present an unusual case presentation of translucent nodule on the upper right cheek of the face which was diagnosed clinically as basal cell carcinoma. Physician should include DF in the differential of such lesions.

## 1 INTRODUCTION

Dermatofibroma (DF) is a common benign skin tumor (Benign Fibrous Histiocytoma) that mostly affects the extremities more than any other site in the body with a tendency to occur more in older females than males.^1^ It usually presents as a slow growing small (<2 cm) brown dome shape papule on the extremities^2^ compression on both sides yield dimple sign, it appears slowly over months and then become stable for years and sometimes regresses spontaneously.^3^ Multiple histologic variants have been described including but not limited to, granular cell dermatofibroma,^4^ clear cell dermatofibroma,^5^ palisading cutaneous fibrous histiocytoma,^6^ aneurysmal,^7^ and cellular.^8^ And dermatoscopic patterns of dermatofibroma have been described in the literature.^9^ Facial involvement of dermatofibroma is considered rare and tends to be more aggressive and hard to treat and could be often misdiagnosed as unusual basal cell carcinoma, adnexal neoplasm or leiomyoma, and cutaneous lymphoma. The presentation can be difficult as it usually presented as firm and ill‐defined.^10^ We here report a clinically atypical case of dermatofibroma developing on the right cheek of the face.

Informed consent was obtained from the patient to be included in the study.

## 2 CASE REPORT

**Figure 1:**
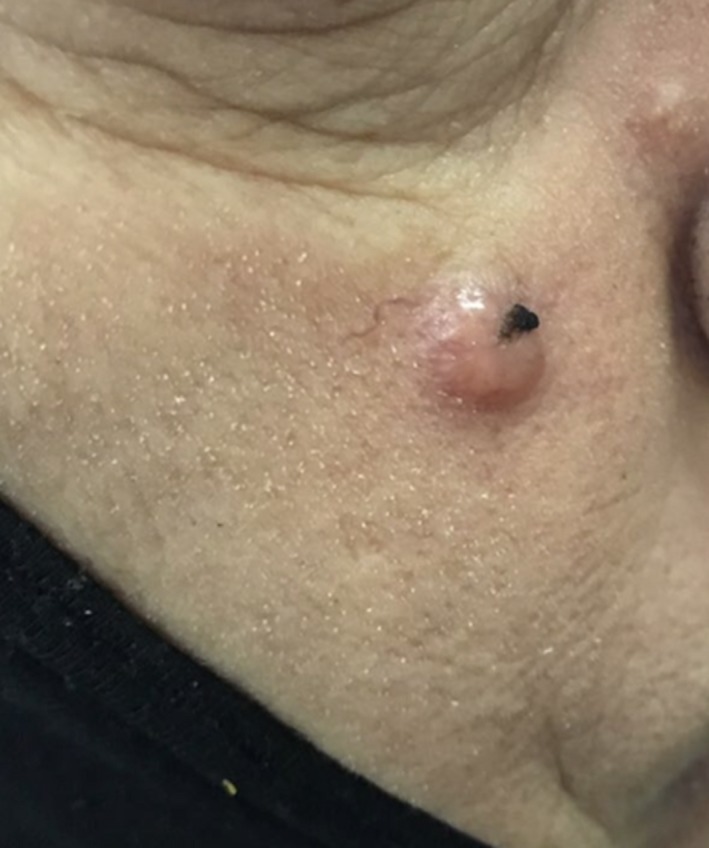
Right upper cheek, 1 cm × 0.5 cm translucent nodule

We present a case of 62‐year‐old lady known to have diabetes type 2, dyslipidemia, hypertension, and history of stroke. She presented to the dermatology clinic with erythematous nodule over the right cheek, it has been present for 2 months. The lesion is single occasionally painful, it has recently increased in size gradually over time and then became stable. Physical examination revealed 1 by 0.5 cm translucent nodule over the right cheek (Figure 1). A punch biopsy was taken.

**Figure 2:**
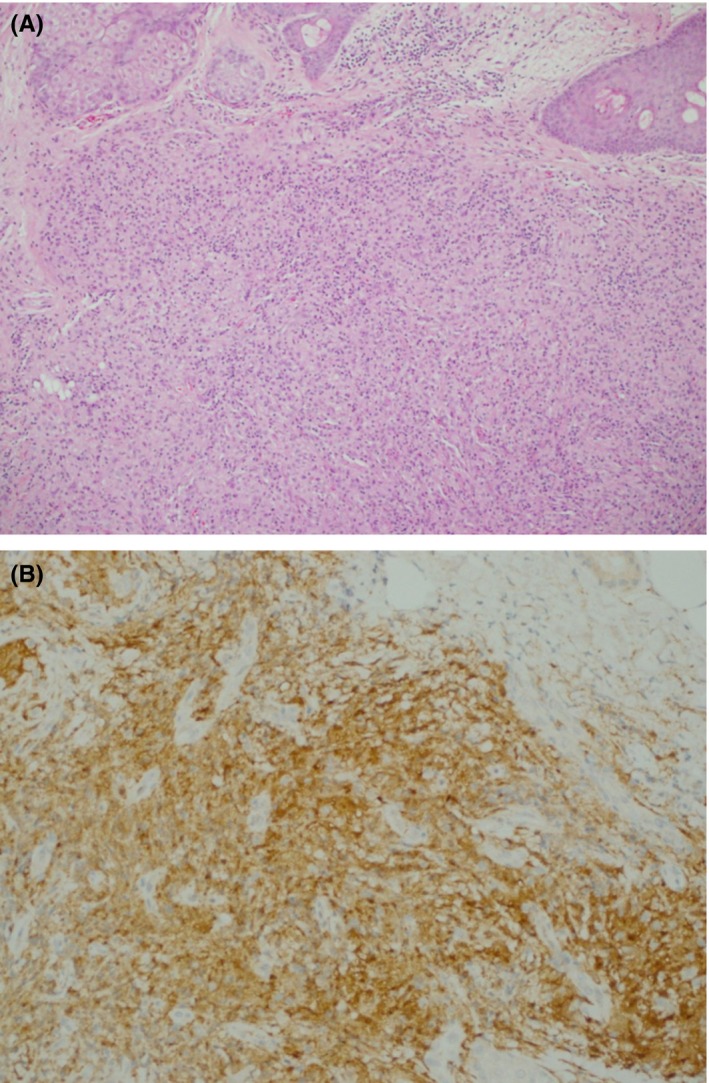
A, Fibroblast‐like spindle cells with focally storiform arrangement (H&E 10x). B, Histiocytic cells stained positively with diffuse CD68 (20 × immunoperoxidase)

The histologic sections reveal nonencapsulated dermal lesion consists of spindle cells have scant cytoplasm, arranged in vague storiform pattern mixed with inflammatory cells including foam cells and lymphocytes (Figure 2A). The cells are reaching focally to subcutaneous tissue and reach to fat with no evidence of tissue destruction. There are no mitotic figures, cellular atypia, nor necrosis.

The immunohistochemistry shows the expression of factor XIII and CD68 (Figure 2B). Other immunostains including pan cytokeratin, CD20, CD34, ALK, CD30, S100, and HMB45 are negative. These findings confirm the diagnosis of benign fibrous histiocytoma (dermatofibroma) and exclude the clinical impression of basal cell carcinoma, amelanotic melanoma, keratoacanthoma, Merkel cell carcinoma, and sebaceous adenoma. The patient has many comorbidities so her family suggested we don't go further at this point, otherwise, the management was planned as excision of the lesion.

## 3 DISCUSSION

Benign Fibrous histiocytoma is considered as one of the most common benign tumors of the skin, and it has very low recurrence rate ranging from 3% to 5%.^11^ Dermatofibroma is mostly asymptomatic and painless. It can develop after a minor trauma or insect bite and predominately occurs in the lower extremities.^12^

There have been other forms of DF in the face that includes oral cavity, eyelid, and scalp. All of which has occurred in deep structures of the subcutaneous skin. It was not diagnosed clinically but rather after the surgical excision and pathological diagnosis that revealed Benign Fibrous Histiocytoma BFH.^13^, ^14^, ^15^

Dermatofibroma (DF) rarely involves the face though there have been a few cases with facial involvement reported in the literature.^10^ Mentzel et al in 2001 described a series of more than thirty thousand dermatofibroma cases only 34 were confined to the facial area specifically involving the Forehead, cheek, eyebrow, ear, and nose. The majority of these cases (17) had an aggressive progression leading to soft tissue and muscle infiltration. While three cases only refined to the dermis. Histologically, only nine cases consisted of absolute typical storiform pattern with the majority showing positive actin spindle‐shaped myofibroblasts and are composed of cellular fascicles. It is recommended to excise the lesions with wide margins.^10^

To the contrary, Estela et al in 2013 have reported 22 cases of dermatofibroma involving the facial area over a course of two decades that did not have any resembling features that may be worrisome. There was no deep tissue involvement except in only three cases. Storiform pattern and spindle cell fascicles histologically with no mitotic activity were observed. No recurrence with minimal excision of the margins was advised.^16^

Dermatofibroma has to be differentiated from dermatofibrosarcoma protuberans which can brutally invade deep structures and produce a higher mitotic rate. In the immunohistochemistry, it stains with CD 34 positively and negatively for factor XIII which are a useful distinguishing characters.^12^

## CONFLICT OF INTEREST

None declared.

## AUTHOR CONTRIBUTION

MQ^1+2^: wrote the initial draft and edited the final manuscript. SK: conceived the idea, care for the patient, and reviewed the final draft. MA: provided the pathology with the description**.**
